# Magnetic resonance imaging in children: common problems and possible solutions for lung and airways imaging

**DOI:** 10.1007/s00247-015-3420-y

**Published:** 2015-09-05

**Authors:** Pierluigi Ciet, Harm A. W. M. Tiddens, Piotr A. Wielopolski, Jim M. Wild, Edward Y. Lee, Giovanni Morana, Maarten H. Lequin

**Affiliations:** Department of Radiology, Sophia Children’s Hospital, Erasmus Medical Center, Rotterdam, The Netherlands; Department of Pediatric Pulmonology and Allergology, Sophia Children’s Hospital, Erasmus Medical Center, Rotterdam, The Netherlands; Academic Radiology, University of Sheffield, Sheffield, UK; Departments of Radiology and Medicine, Pulmonary Divisions, Boston Children’s Hospital and Harvard Medical School, Boston, MA USA; Department of Radiology, Ca’ Foncello Regional Hospital, Treviso, Italy; Department of Radiology, Wilhelmina Children’s Hospital, University Medical Center, Wilhelmina Kinderziekenhuis (WKZ) Lundlaan 6, 3584 EA Utrecht, The Netherlands

**Keywords:** Lung, Airways, Magnetic resonance imaging, Computed tomography, Child, Technique

## Abstract

Pediatric chest MRI is challenging. High-resolution scans of the lungs and airways are compromised by long imaging times, low lung proton density and motion. Low signal is a problem of normal lung. Lung abnormalities commonly cause increased signal intenstities. Among the most important factors for a successful MRI is patient cooperation, so the long acquisition times make patient preparation crucial. Children usually have problems with long breath-holds and with the concept of quiet breathing. Young children are even more challenging because of higher cardiac and respiratory rates giving motion blurring. For these reasons, CT has often been preferred over MRI for chest pediatric imaging. Despite its drawbacks, MRI also has advantages over CT, which justifies its further development and clinical use. The most important advantage is the absence of ionizing radiation, which allows frequent scanning for short- and long-term follow-up studies of chronic diseases. Moreover, MRI allows assessment of functional aspects of the chest, such as lung perfusion and ventilation, or airways and diaphragm mechanics. In this review, we describe the most common MRI acquisition techniques on the verge of clinical translation, their problems and the possible solutions to make chest MRI feasible in children.

## Introduction

Chest MRI is challenging because of the magnetic heterogeneous environment in the chest region [1]. Lung parenchyma is a low proton density structure and hence has a reduced signal-to-noise ratio [2]. In addition, the numerous air-tissue interfaces within a voxel induce strong localized microscopic magnetic field gradients, which produce extensive MRI signal dephasing leading to extremely short T2 star (T2*) and geometric distortions. These effects become stronger at higher magnetic field strengths (i.e. 3 T), which are increasingly used in clinical settings for enhanced signal-to-noise ratio [3]. However, signal-to-noise ratio in cases of lung pathology, such as pneumonia, edema, tumors and atelectasis, is increased by higher fluid content and amount of tissue. These conditions result in higher proton density and improved visualization [4].

Longer scan times with MRI lead to respiratory and cardiac motion artifacts. Respiratory artifacts can be reduced by using breath-hold acquisitions, which can provide better image quality despite lower resolution [5]. Unfortunately, many lung diseases result in increased respiratory rates and maintaining breath-holds of several seconds may become impossible [5]. This problem is even bigger in young children and infants, who have a higher respiratory rate than adults [5], and where cooperation is often not possible. Thus, many well-established MRI techniques used in routine studies of brain, neck and peripheral vasculature cannot be easily implemented in the chest region due to inconsistent image quality.

To address this problem, an MRI sequence with high temporal resolution and diagnostic level signal-to-noise ratio is required. MR physicists have worked hard to develop techniques that meet these challenging criteria [6, 7]. The main driving force to develop sophisticated MRI sequences for pediatric chest imaging is that MRI is a radiation-free technique. This is especially important for children who are more sensitive to ionizing radiation than adults [8]. This justifies the use of chest MRI for short- and long-term follow-up of chronic lung diseases such as cystic fibrosis, so reducing the lifelong cumulative radiation dose [3]. Moreover, MRI has the advantage of integrating anatomical and functional information in a single examination, a possibility not as readily available with other imaging modalities. MRI can provide functional information regarding lung perfusion using gadolinium contrast [9], lung mechanics using dynamic acquisitions [10], and ventilation using inhaled hyperpolarized gases [11], oxygen enhancement or dynamic motion-based methods [12].

For all the aforementioned reasons, MRI has been introduced in clinical practice, but its use on a large scale is still far off. In this article, we review the challenges currently faced in chest MRI and the most common techniques used for imaging lung and airways diseases in children.

## Patient cooperation

Image quality in chest MRI depends on patient cooperation, which is in turn related to patient age, and mental and disease status [5]. Children with dyspnea, younger than 6 years, mentally impaired or with hearing problems might not be able to follow breathing instructions. In these cases, even the use of free-breathing MRI techniques, which are described below, cannot overcome patient motion, making the use of sedation inevitable. Under sedation child’s breathing is guided by the anesthesiologist, who can safely reproduce breath-hold conditions under continuous monitoring of a child’s vital signs. The radiologist is responsible for keeping the MRI scan as short as possible, limiting anesthesia time and preventing development of atelectasis. Nevertheless, a risk-to-benefit assessment of sedation, imaging modality and exam indication is crucial to obtain diagnostic scans and prevent long-term sequelae in infants and young children [13].

**Fig. 1:**
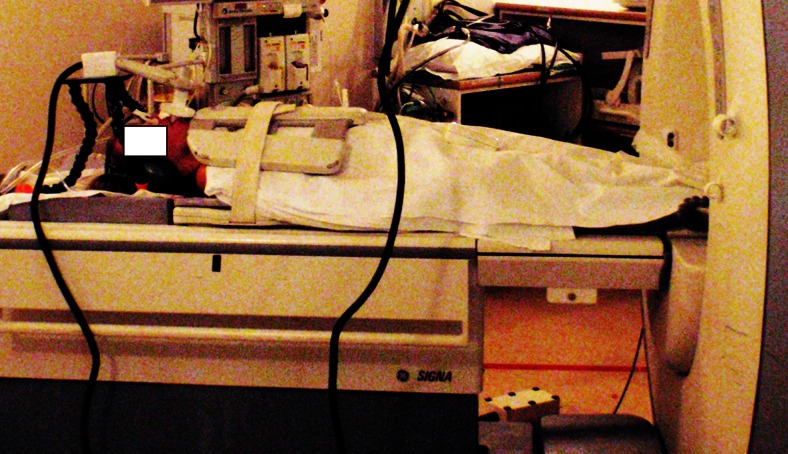
MR compatible spirometer setting during MRI of a 10-year-old girl with cystic fibrosis. A plastic adjustable tripod holds the mouthpiece that is connected through a plastic black corrugated tube to the control computer at the scanner operator’s side. The child is in the feet-first position to ease the use of the mouthpiece in the MRI gantry

In children older than 6 years, cooperation can be increased substantially with adequate training in a mock scanner and by using a MR-compatible spirometer [10]. Typical training consists of rehearsing specific breathing maneuvers inside the mock scanner before the scan is performed. Training can be performed in the supine position adopted in the MRI scanner on a bed using a MRI-compatible spirometer (Fig. 1). In our institution, this training is performed by dedicated lung function technicians and lasts on average 15–30 min. The four main purposes of this training are: 1) to obtain inspiratory and expiratory capacity in supine position as a point of reference for the actual scanning, 2) to train the child to execute specific breathing maneuvers during the MRI, 3) to reduce anxiety related to MRI investigations and 4) to increase the number of successful MRI investigations [10]. Based on our experience, more than 90% of the children successfully complete the MRI examination after training with the MRI-compatible spirometer. Commonly used breathing maneuvers are: breath-hold at full inspiration or expiration, forced expiration after full inspiration, coughing, and quiet free and quiet deep inspiration-expiration breathing. These maneuvers allow the study of the chest anatomy or the response of the airways to different air flow conditions [10].

## MR system and coil selection

Appropriate MRI system selection is crucial because it strongly influences the overall image quality in chest MRI. Theoretically, signal-to-noise ratio would be enhanced, like in other imaging regions [4], by using higher magnetic field strengths, but this is not the case for chest MRI. For lung MRI, higher field strength does not result in higher signal-to-noise ratio due to the increase in T2* dephasing (between 1 ms and 2 ms at 1.5 T and 0.5 ms and 1 ms at 3 T) and susceptibility artifacts. For this reason, 1.5-T systems are currently more suitable for routine clinical lung MRI than 3-T systems.

Making the correct coil selection for chest MRI is another critical factor that determines image quality. Close fitting receiver array coils are of key importance [14]. Array coils provide higher signal-to-noise ratio by virtue of the closer proximity to the lungs but also allow for shorter acquisition time [14] through the use of parallel imaging [6]. Optimal coil designs differ depending on the anatomy; for thoracic MRI, the coils most frequently used are arrays with 8 to 32 channels.

## Lung morphology and image quality

Inherent low image quality of chest MRI results from: a) low proton density of normal lung tissue, b) presence of a large number of air-tissue interfaces, and c) artifacts from breathing and cardiovascular pulsations during data collection. In the sections below, these points are clarified.

### Low proton density of the lung and signal optimization

Lung MRI has an intrinsic low signal-to-noise ratio because air does not provide any MR signal and the number of water protons present in lung parenchyma is low. This is not the case for central airways or vascular structures, which can be visualized quite easily [15].

A compromise between spatial resolution and acquisition time must be made in order to obtain the desired image quality in reasonable examination times [3].

In chest MRI, increasing the voxel size proportionally augments the signal-to-noise ratio because it increases the number of water protons per voxel imaged [16]. Consequently, in chest MRI, the voxel size is set larger than CT [3]. Hence, thicker slices are acquired (between 2 mm and 8 mm) and spatial resolution is lowered to shorten acquisition time [17].

**Fig. 2:**
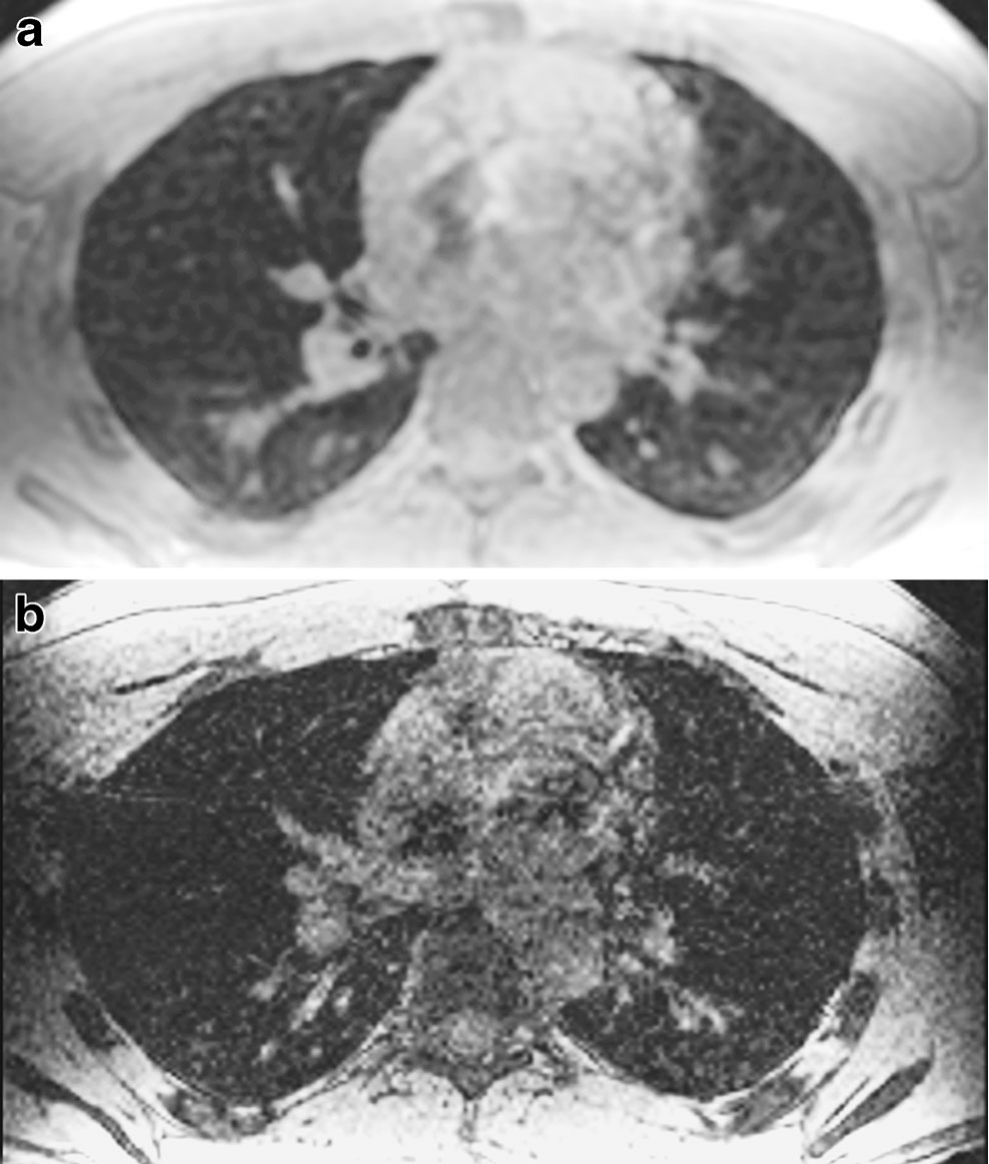
Effect of voxel size on signal-to-noise ratio at 3 T. Axial reformat from a sagittal 3-D spoiled gradient echo acquisition with isotropic voxels of (**a**) 3.0 × 3.0 × 3.0 mm^3^ (3 s scan time) and (**b**) 1.5 × 1.5 × 1.5 mm^3^ (14 s scan time) in a 28-year-old male volunteer. By halving each dimension, the signal-to-noise ratio is reduced by a factor of 8; note the increased image noise in (**b**)

To retain the same acquisition time, increasing the field-of-view augments signal-to-noise ratio because it translates into larger voxel sizes [16] Keeping the same field of view and coverage with smaller voxel sizes can decrease signal-to-noise ratio dramatically (Fig. 2). Typically, field of view is selected according to patient size and in chest MRI is usually large in order to get good signal-to-noise ratio. For children, care must be taken not to select too small a field of view and voxel size for not running out of signal-to-noise ratio.

**Fig. 3:**
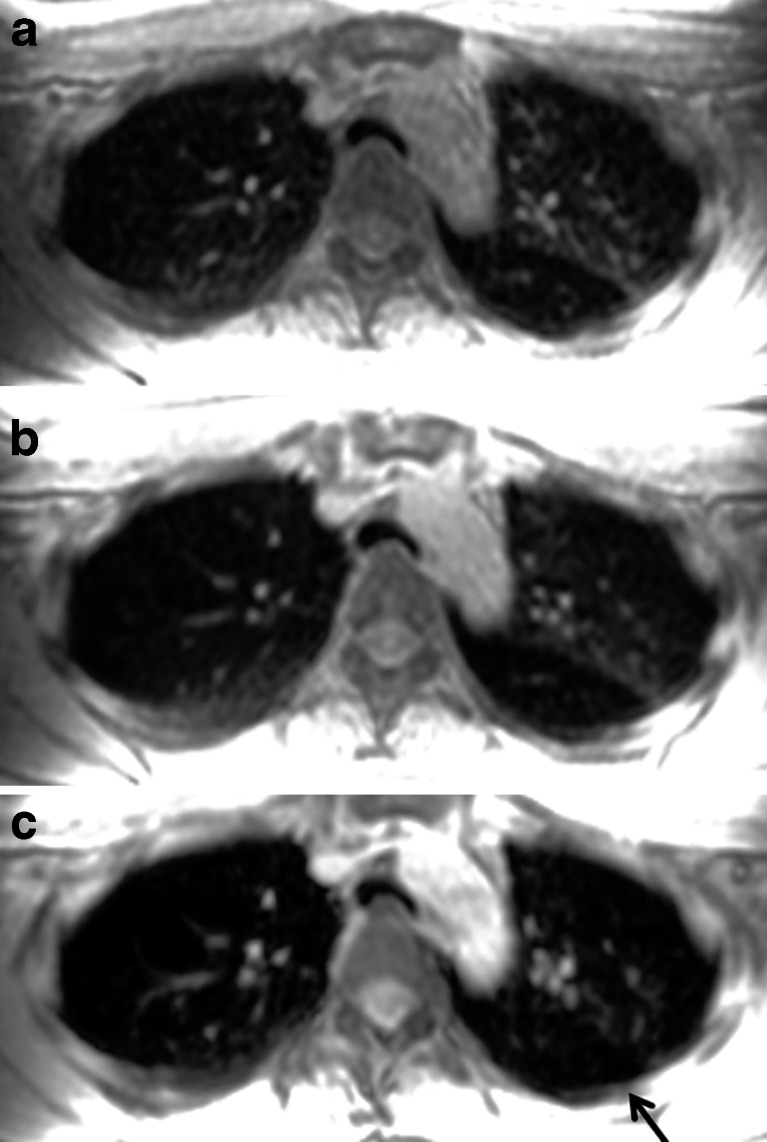
Effect of bandwidth on signal-to-noise ratio. Three-dimensional spoiled gradient echo axial reformat with a readout bandwidth of (**a**) 142 kHz, (**b**) 62.5 kHz and (**c**) 31.25 kHz in an 18-year-old girl with asthma. Note that lower bandwidth reduces the lung parenchyma signal because of longer echo time, as shown in (**c**), where the area of trapped air (*arrow*) in the left lower lobe is less clearly identified than in (**a**)

The receiver bandwidth is the range of frequencies collected by an MRI system during frequency encoding [16]. The MR data acquisition can take more or less time, depending on the bandwidth. Halving bandwidth improves signal-to-noise ratio by 41%. On the other hand, with a lower bandwidth, there will be more chemical shift and motion artifacts [15]. Therefore, a lower bandwidth may not be beneficial for lung imaging, especially for gradient echo sequences, because it enhances the sensitivity to motion and field inhomogeneity artifacts and may increase breath-hold times [16] (Fig. 3).

Signal-to-noise ratio loss is also factored if partial Fourier scanning or parallel imaging are used [6, 18]. Partial Fourier imaging is used to reduce scan time in chest MRI. Underling k space lowers the signal-to-noise ratio by as similar proportion, e.g., a half-Fourier scan almost halves the signal-to-noise ratio [18].

Similarly, scan time reduction achieved through parallel imaging techniques influences signal-to-noise ratio. All parallel imaging techniques reduce acquisition time according to the acceleration factor selected by the operator, which usually ranges from 2 to 4 times for 2-D and from 2 to 16 times for 3-D imaging. Acceleration factors higher than 2 for 2-D imaging are seldom used [17]. In fact, the higher the accelerator factor, the higher the penalty in signal-to-noise ratio. However, higher acceleration factors can be selected when diseased states are present in the chest (i.e. tumor), or larger and thicker airways (i.e. bronchiectasis) or when contrast agents are used to shorten the T1 of lung parenchyma, blood or surrounding structures. Finally, the main benefits of using partial Fourier and parallel imaging is to freeze motion and eliminate the detrimental effects of breathing by using breath-hold acquisitions [4].

### Respiratory and cardiac motion

**Fig. 4:**
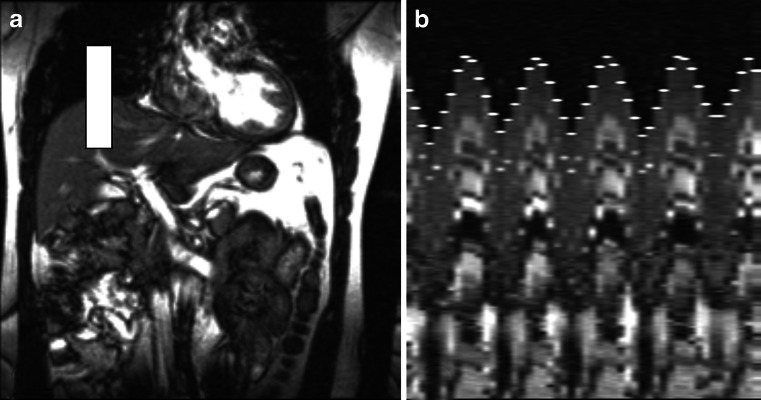
Navigator-based acquisition. **a** An excitation pencil is placed on the diaphragmatic dome at an end-expiration localizer (*white box*). **b** Respiratory waveform is reconstructed in a 35-year-old male volunteer

When patients cannot execute breath-hold maneuvers, free-breathing acquisitions can be considered using navigator echoes or pneumobelts. When using navigator echoes, the diaphragm position is detected in real time to trigger the image acquisition prospectively by selecting the position of the diaphragm during expiration. The position in expiration is based on a short observation period at the beginning of the acquisition, during which a respiratory waveform is reconstructed from a series of breathing cycles [19] (Fig. 4). Navigator echo-based techniques dramatically improve image quality without the need for patient cooperation but at the expense of longer acquisition times. Acquisition time highly depends on the regularity of the breathing pattern when using a prospective data acquisition scheme [19]. This technique has been used in children as young as 4 years old without sedation [20].

**Fig. 5:**
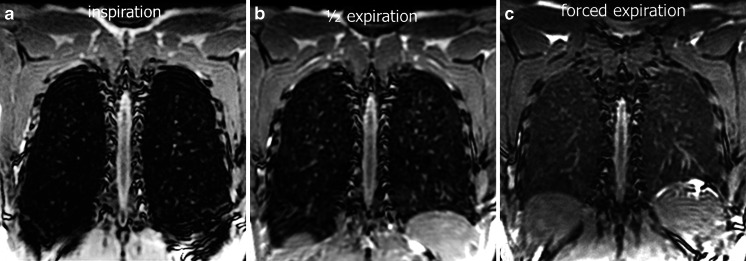
Effect of inspiratory level on lung signal-to-noise ratio using 3-D steady-state free precession. Coronal reformats at (**a**) end-inspiration, (**b**) half-expiration and (**c**) forced expiration in a 28-year-old male volunteer. Note the progressive increased signal-to-noise ratio of the lung parenchyma from (**a**) to (**c**)

Respiratory gating can also be obtained using pneumobelts or similar external respiratory devices to trigger the scan prospectively [21]. A pneumobelt consists of a flexible corrugated tube placed around the rib cage or the abdomen, with the selected position depending on the maximum distention recorded during the respiratory cycle. Similar to the navigator echo system, image acquisition is mostly performed during expiration because it is more reproducible compared to inspiration. However, scanning lungs in inspiration will result in more problems than scanning during expiration, when lung density is higher due to reduced air content (Fig. 5).

Another established method to overcome breathing motion is by averaging multiple acquisitions during free breathing. This method leads to image blurring but reduces image ghosting [22]. In general, this technique increases the acquisition time but mostly without increasing total MRI scanning time.

To avoid blurring and ghosting artifacts from cardiac motion, electrocardiogram (ECG) gating can be additionally incorporated [23]. ECG gating is not important for the upper portion of the lung, such as the apices, but it becomes more important for the diagnosis of pathologies involving the right middle lobe, lingula and the left lower lobe. The use of ECG and respiratory triggering together in general increases scan time substantially as imaging data is only accepted if both respiratory (at expiration) and cardiac triggering (at diastole) coincide.

## MRI pulse sequences for chest MRI

Sequences for chest MRI should be selected based on type of contrast required (T1-weighted, T2-weighted or proton density), signal-to-noise ratio, contrast-to-noise ratio, tissue characterization, and spatial and temporal resolution. Here, we summarize spin echo and gradient recalled echo based MRI sequences from different vendors used to collect 2-D and 3-D images during breath-hold, which are usually preferred for chest MRI [3].

### Fast spin echo

Fast spin echo sequences have the same low sensitivity to magnetic susceptibility artifacts as spin echo and can be also used with sub-second acquisition times (e.g., single-shot fast spin echo) [24]. A typically robust sequence is the 2-D T2-weighted single-shot fast spin echo, known under different acronyms depending on the MRI scan manufacturer [25]. Single-shot fast spin echo techniques have high sensitivity and high signal-to-noise ratio for fluid detection.

**Fig. 6:**
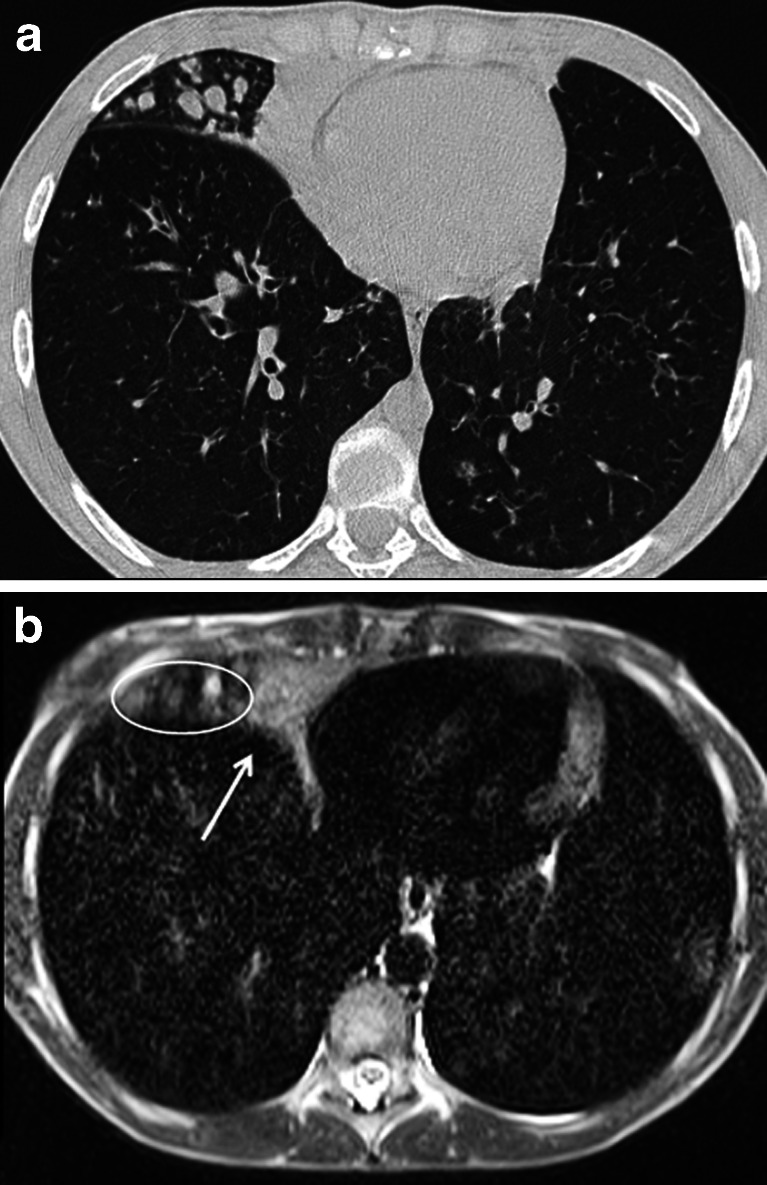
Steady-state free precession MRI in a 12-year-old girl with cystic fibrosis. Comparison between (**a**) CT and (**b**) MRI at end-inspiration. Note the consolidation in the right middle lobe (*arrow*) and the adjacent mucus-filled bronchi (*circle*)

The clinical utility of single-shot fast spin echo scans for lung imaging has been shown in different studies for the detection of pulmonary pathologies, such as pneumonia in immune-compromised patients [26, 27]. In a study at low magnetic field strengths, single-shot fast spin echo was the preferred sequence for the visualization of mediastinum and lung consolidations [28]. Single-shot fast spin echo is also useful to assess pneumonia complications, namely abscess, necrosis and empyema. Moreover, these T2-weighted sequences are indicated in those cystic diseases with increased water content, like congenital pulmonary airway malformations and cystic fibrosis [5]. In cystic fibrosis, this sequence delineates easily bronchiectasis and mucus plugs, which both have high signals in T2-weighted imaging (Fig. 6).

**Fig. 7:**
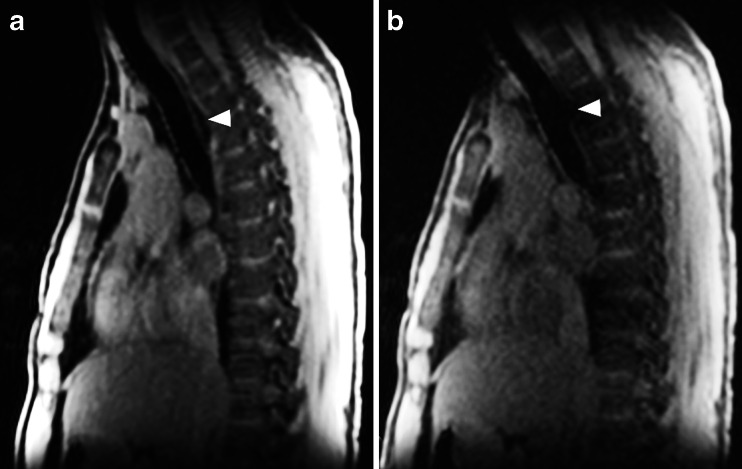
Fat-suppression techniques. **a** Dixon fat attenuation versus (**b**) spectral fat saturation in a 35-year-old volunteer. Note more homogenous fat signal suppression with the Dixon technique, the spectral fat saturation being more influenced by magnetic field heterogeneity and suppressing the tracheal signal (*arrowhead*)

Fat saturation techniques are commonly used in chest MRI to suppress or detect the signal from adipose tissue. These techniques are usually combined to fast spin echo sequences to enhance fluid detection (i.e. pleural effusion) or with gradient echo sequences after contrast administration to enhance tumor detection. Fat saturation can be also achieved using short-tau inversion recovery (STIR) preparations [29]. Although the saturation technique is faster, it is less reliable than STIR because of magnetic field inhomogeneities of lung parenchyma and across the chest region. On the other hand, the inversion radiofrequency pulse used in STIR for fat signal cancellation lowers the overall signal-to-noise ratio [29]. Finally, Dixon-based fat separation technique, a fast 3-D gradient echo sequence, can provide a more homogeneous removal of lipid signals than conventional frequency selective chemical saturation techniques [29] (Fig. 7).

### Gradient recalled echo

All variations of 2-D and 3-D short and ultra-short echo time gradient recalled echo sequences are typically considered the most robust sequence for chest MRI [4]. These scans when used with short and ultra-short echo times can overcome the short T2* of lung parenchyma and minimize the signal loss created by the extensive air-tissue interfaces. Gradient echo sequences are usually collected with very short repetition times. A short repetition time is critical for patients who are unable to accomplish long breath-holds (>10 s) [3].

**Fig. 8:**
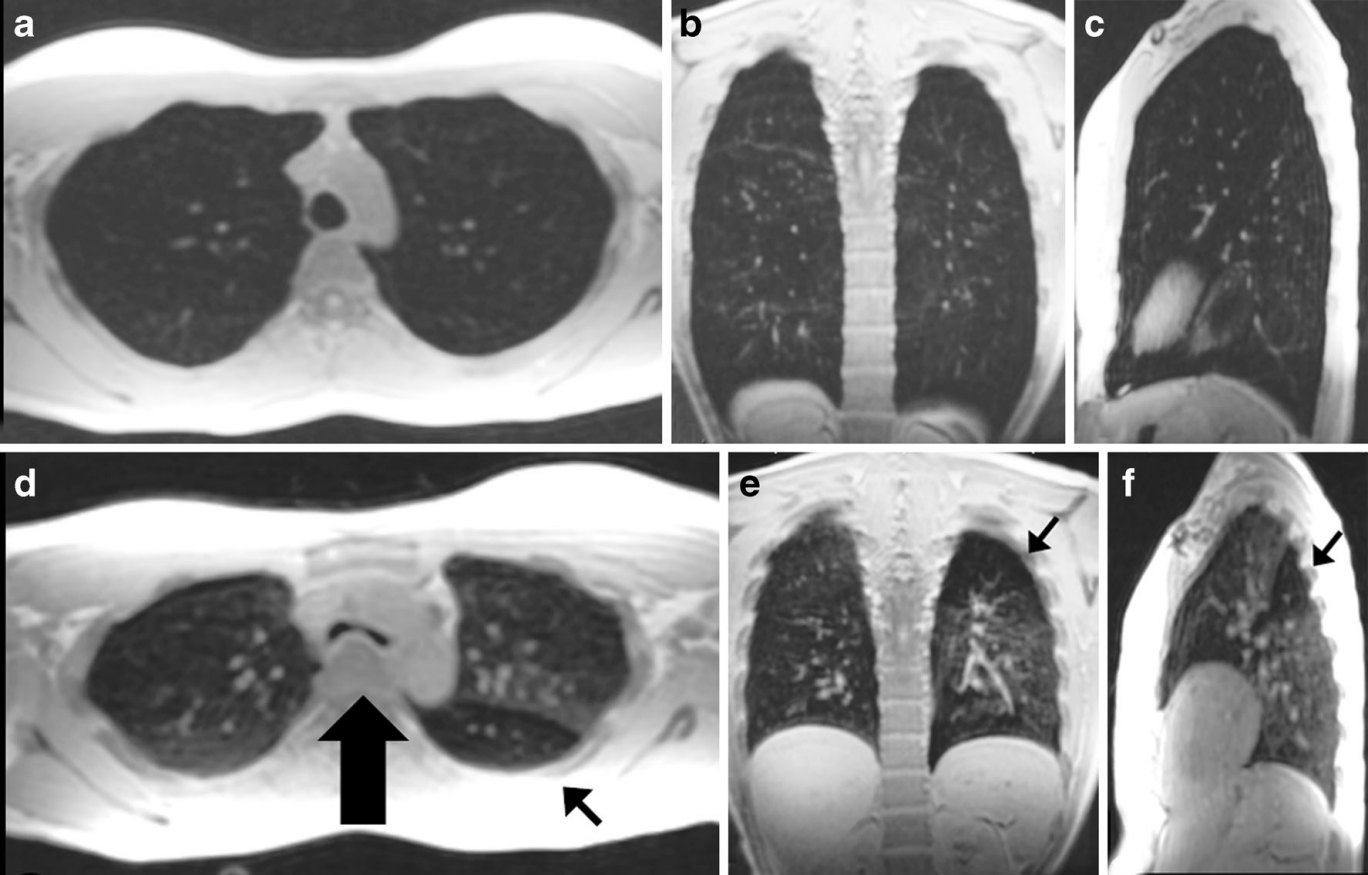
Three-dimensional spoiled gradient echo sagittal acquisition with axial and coronal reformats at (**a**–**c**) end-inspiration and (**d**–**f**) end-expiration in a 28-year-old female asthma patient. Note area of air trapping (*thin arrow*) in the left lower lobe with excessive collapse of trachea in the expiratory image (*thick arrow*). Acquisition time of 6 s with isotropic voxels of 3.0 × 3.0 × 3.0 mm^3^

In general, 3-D gradient echo acquisitions are preferred over 2-D scans because they can provide better signal-to-noise ratio and are less sensitive to susceptibility artifacts. Overall, 3-D gradient echo sequences can acquire the entire thorax with adequate resolution and signal-to-noise ratio (e.g., isotropic voxels ≥3x3x3 mm^3^ in less than 15 s) [10]. When isotropic voxels are collected, multiplanar reformats can be performed in any orientation, which is crucial in the evaluation of vascular and airways structures [4] (Fig. 8). There are two types of gradient echo sequences: spoiled gradient echo and steady-state free precession sequences.

For a fixed repetition time, spoiled gradient echo sequences provide contrasts ranging from proton-density weighted (at low flip angle) to T1 weighted (at high flip angle). The former is mostly used to investigate lung parenchyma and airways without the use of contrast agents, while the latter is used to assess vascular structures and lung parenchymal perfusion after contrast administration.

**Fig. 9:**
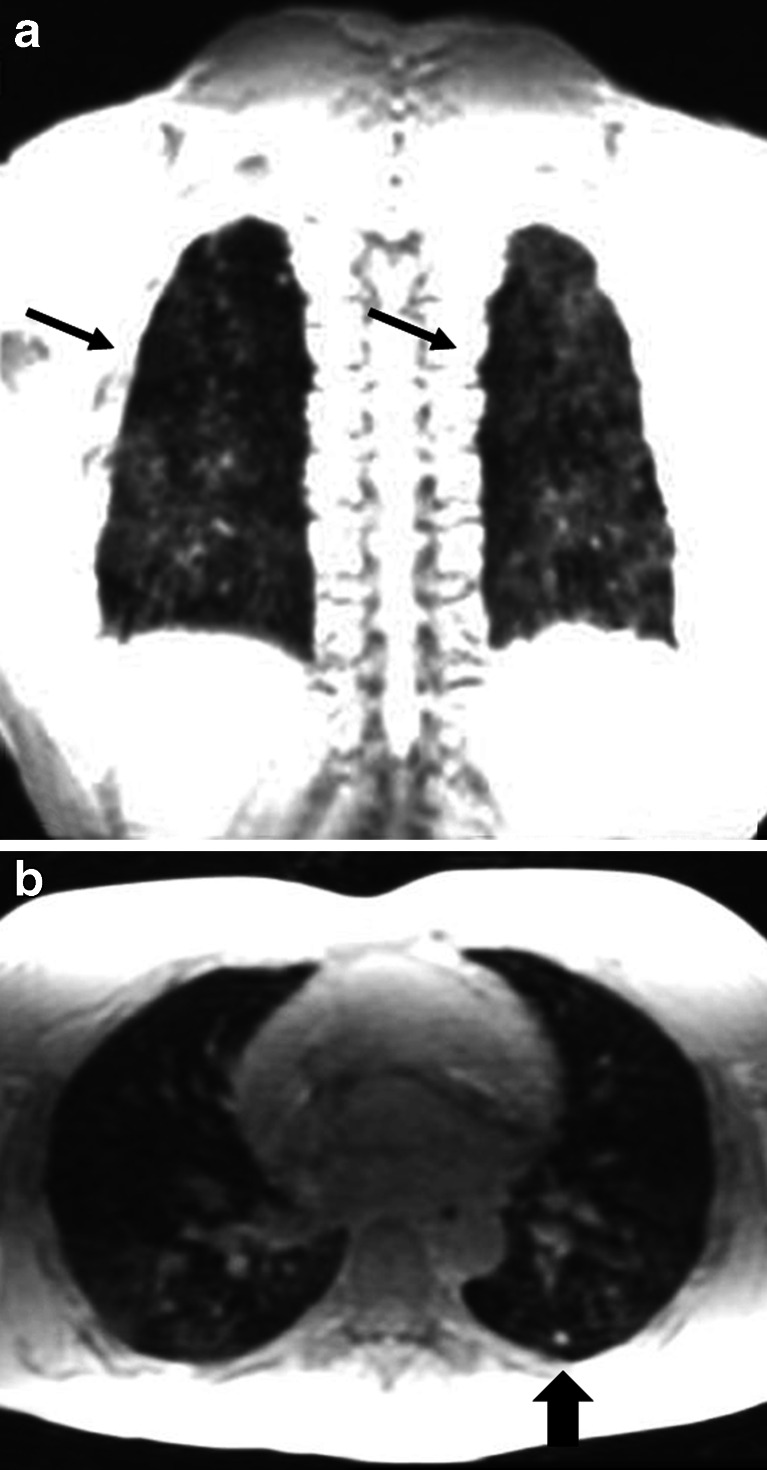
End-expiratory 3-D spoiled gradient echo. **a** Coronal and (**b**) axial reformats acquired at 1.5 T in a 22-year-old man with influenza symptoms. Note areas of air trapping (*thin arrows*) and left lower lobe subpleural nodule (*thick arrow*)

Spoiled gradient echo sequences have been used in several clinical studies, for instance it was rated as the best sequence for lung nodule detection in vivo [28] (Fig. 9). In another comparative study in a porcine chest phantom, this sequence had similar diagnostic accuracy as CT in depicting pulmonary nodules larger than 8 mm, and sensitivity between 80% and 90% for lesions larger than 4 mm [30]. Lesions smaller than 4 mm were difficult to detect with MRI [30]. Two-dimensional and 3-D spoiled gradient echo scans are also the preferred choice for contrast-enhanced studies, where contrast-enhancing lesions, vessels and lymph nodes can be better delineated after gadolinium injection [3] and dynamic contrast-enhanced perfusion imaging of the lungs.

The second group of sequences (steady-state free precession) generates a T2/T1-weighted contrast with medium to high flip angle settings (>30°) using a very short repetition time, so enhancing tissues in the lung with more water-like behavior, such as mucus plugs in the airways, have high signal intensities [16]. Two-dimensional steady-state free precession scans are frequently used because they allow fast acquisitions of the entire thorax in a single breath-hold with good signal-to-noise ratio. At the same time, this type of sequence can be rather sensitive to magnetic field inhomogeneities, which become quite problematic at higher magnetic field strengths (i.e. 3 T). These inhomogeneities result in off-resonance banding artifacts, which are usually seen above the diaphragm [16]. These artifacts tend to alter the lung signal intensity, so signal from nodules and vessels are subsequently cancelled [31]. The artifacts can be reduced by substantially shortening the repetition time [31]. Another limitation of steady-state sequence is that it has an intensive specific absorption ratio, which with high flip angles and at higher field strengths can exceed the maximal safety level allowed in MRI.

**Fig. 10:**
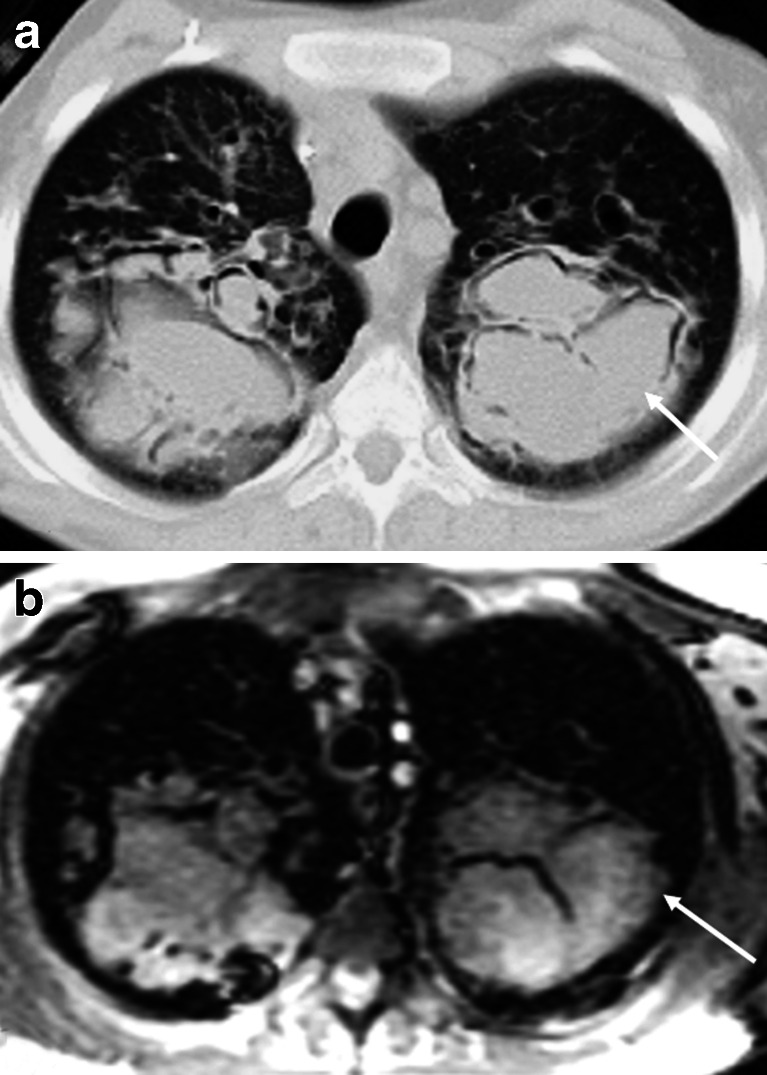
Aspergilloma in cystic fibrosis. **a** CT versus (**b**) steady-state free precession MRI in a 16-year-old boy with end-stage cystic fibrosis. Note widely dilated bronchi filled with thick mucus and aspergillomas (*arrows*)

**Fig. 11:**
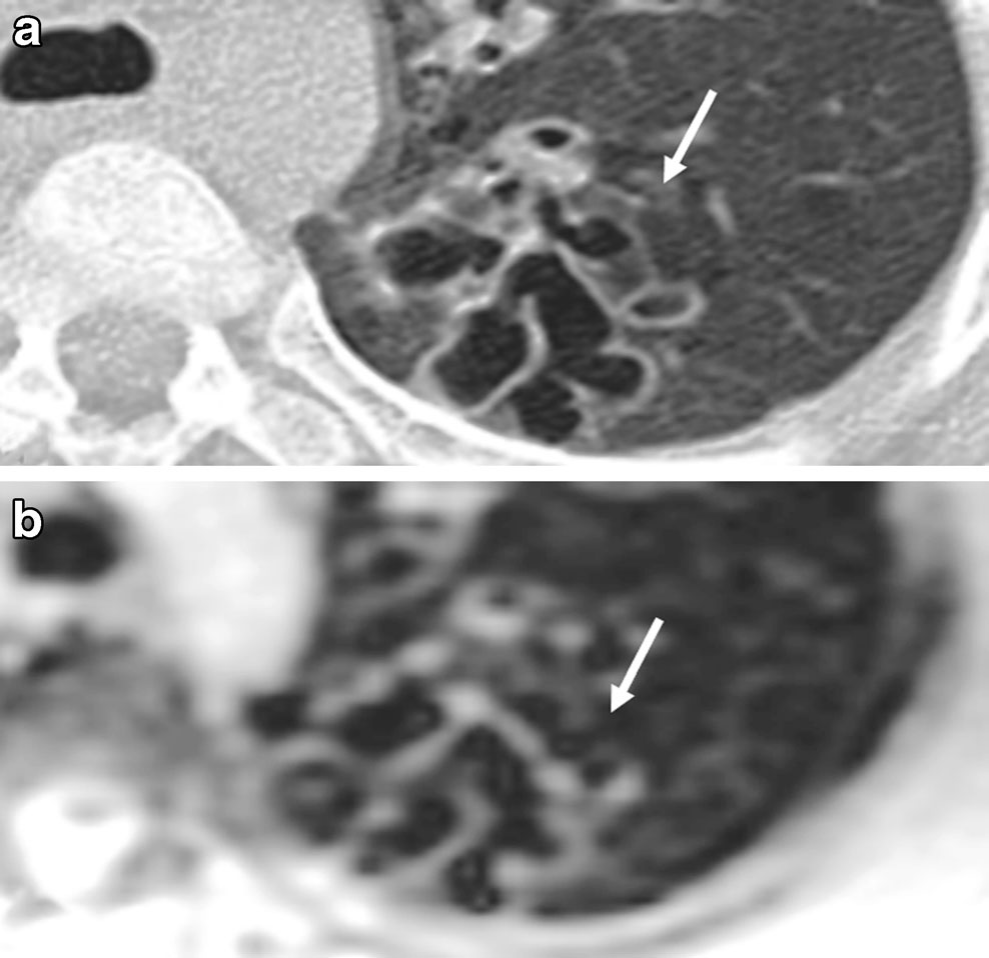
Bronchiectasis. **a** CT versus (**b**) steady-state free precession MRI in a 14-year-old boy with cystic fibrosis demonstrate bronchiectasis in the left upper lobe (*arrows*)

Steady-state free precession sequences are commonly used in chest MRI. In a comparative study between different MRI sequences, it was the preferred sequence to visualize lung parenchyma in volunteers with the fewest motion artifacts [28]. In cystic fibrosis, we showed that steady-state free precession sequences are a sensitive technique for detecting clinically relevant structural abnormalities [32] (Figs. 10 and 11). Since then, we introduced an MRI protocol mainly composed of this sequence, as biennial MRI scans (alternating with CT) to monitor progression of CF lung disease. In children with suspicious pneumonia, steady-state free precession was compared to chest radiograph [33], showing really good correlation between MRI and chest radiograph for all pathological findings [33]. More recently, steady-state free precession scans have been proposed as the best option to assess lung fibrosis [31].

## Projection acquisition and reconstruction techniques

**Fig. 12:**
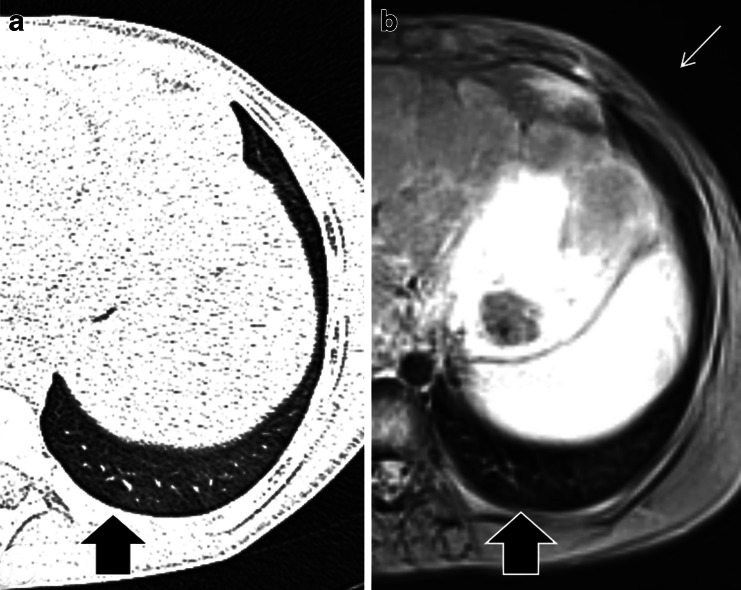
Radial k space-filling (PROPELLER) artifact and trapped air assessment. **a** CT versus (**b**) free-breathing spin echo MRI with radial k space trajectory in a 24-year-old woman with cystic fibrosis. End-expiration scans performed on the same day. Note streak artifacts outside the chest (*thin arrow*) due to the helicoidal k-space reconstruction. Note also area of trapped air in the left lower lobe (*thick arrow*s)

Motion corrected fast spin-echo sequence (i.e. PROPELLER) is a free-breathing sequence frequently used in chest MRI. This free-breathing sequence collects data using rotating k-space bands or blades, which have proven more robust to suppress respiratory motion artifacts [34]. In fact, image acquisition can be combined with respiratory-gated techniques to further reduce the effects of motion. This combined approach allows full chest coverage in 4–7 min, depending on the breathing pattern of the patient, with good in-plane spatial resolution (1–1.5 mm) [3]. Unfortunately, studies have shown that this sequence frequently produces streak artifacts [5] in the lung parenchyma (Fig. 12). To reduce this artifact, the blade width (k-space coverage acquired per blade) should be increased or a finer angular sampling used [35] even at the expense of imaging time.

**Fig. 13:**
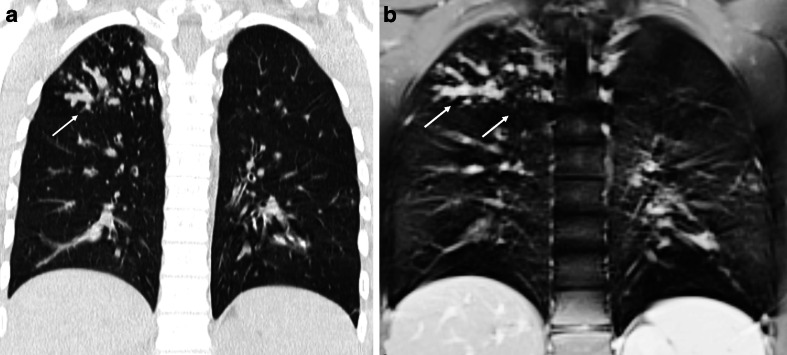
MRI with radial k space trajectory in cystic fibrosis. **a** CT versus (**b**) coronal free-breathing MRI with radial k space trajectory (PROPELLER) in a 21-year-old man with cystic fibrosis. There are areas of mucus plugging and bronchiectasis in the right upper lobe (*arrows*)

In a comparative study with chest radiography, motion-corrected fast spin echo was superior to detect lung abnormalities in patient with middle lobe syndrome [20] (Fig. 13). In patients with common variable immunodeficiency, motion-corrected fast spin echo was proposed as an alternative method to reduce cumulative dose due to repeated CT imaging [36].

## Functional imaging

MRI offers numerous techniques to study different functional aspects of lung and airways. Gadolinium contrast can be used to assess the pulmonary vasculature and lung perfusion [37, 38]. The high temporal resolution of MRI proved to be useful to assess lung and central airway mechanics [10]. Hyperpolarized gas MRI with helium (^3^He) or Xenon (^129^Xe) produces high-contrast images of lung ventilation [5, 11]. Finally, Fourier Decomposition, can be used to assess lung perfusion and ventilation without the use of contrast media [39]. These techniques will be briefly described starting with the clinical available to the most experimental technique.

## Pulmonary magnetic resonance angiography

Magnetic resonance angiography (MR angiography) of the chest can be obtained with or without the intravenous administration of contrast. In both cases, the low proton density of the lungs is ideal for generating high angiographic contrast with all gradient echo sequence types.

### Non-contrast MR angiography acquisitions

**Fig. 14:**
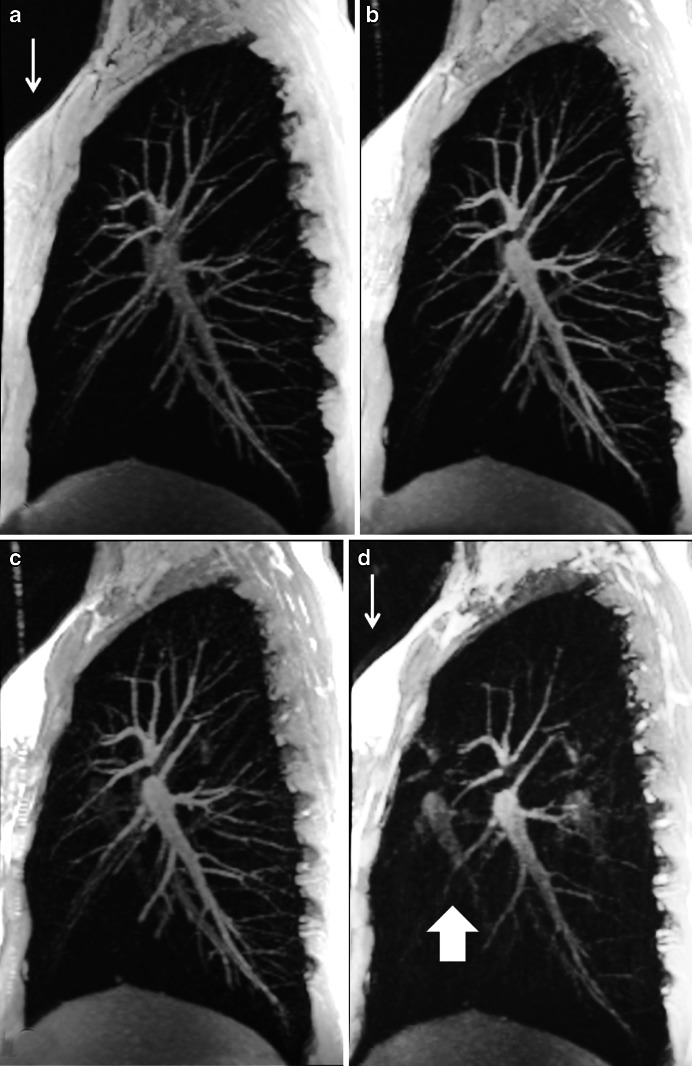
Time-of-flight angiography spoiled gradient echo in a 35-year-old male volunteer, acquired with increasing excitation flip angles: (**a**) 2°, (**b**) 6°, (**c**) 8° and (**d**) 12°. Note that by augmenting the flip angle the contrast shifts from proton density to T1-weighted, as observed on the fat signal intensity increase in the anterior chest wall (*arrows*). Higher flip angles provide better differentiation between arterial and venous signal at the expense of ghosting artifacts (*thick arrow*)

The main advantage of these techniques is that they can be repeated without concerns related to the use of contrast, especially in patients with impaired renal function [5]. When using spoiled gradient echo sequence, MR angiography can be obtained using a proton density weighted contrast or employing the time-of-flight signal enhancement technique [16]. Three-dimensional angiograms can be generated using this time-of-flight technique and using maximum intensity projections or volume rendering can make vessels appear similar to conventional X-ray angiography [16] (Fig. 14). Time-of-flight techniques allow good depiction of arterial vascular morphology whenever inflow effects are adequate (high flow velocities). Different acquisition strategies can be used to perform time-of-flight 2-D and 3-D (Table 1). Pulmonary embolism is best depicted using 2-D or small slab 3-D sequential time-of-flight sequences.

**Table 1:** Characteristics of commonly used versions of time-of-flight MR angiography. These are spoiled gradient echo sequences acquired during breath-hold

Version	Characteristics
2-D or thin-slab 3-D sequential acquisitions	Short TR (5–10 ms), short TE (1–3 ms), flip angle >45°. Tracking saturation bands for selective imaging of pulmonary arteries or veins (mostly for sagittal acquisitions) can be used.
2-D interleaved acquisitions	Long TR (100–280 ms), short TE (1–3 ms), intermediate flip angles (25°–45°). No tracking saturation bands. Inflow effects on the arterial side can provide higher signal than veins.
2-D segmented k-space acquisition with electrocardiographic triggering	Short TR (5–10 ms), short TE (1–3 ms). Optimal inflow effect at peak systole allows higher signal-to-noise ratio and distinction against veins. Can be performed in a cine loop. Usually one thick slice per breath-hold is acquired.
Thick-slab 3-D acquisition	Short TR (5–10 ms), short TE (1–3 ms). Sagittal slab, one lung. Low flip angles or spatially variable RF (TONE, VUSE). Arteries can be seen with higher intensity as compared to veins.

*TE* echo time, *TR* repetition time, *TONE* tilt optimized non-saturated excitation, *VUSE* variable angle uniform signal excitation

**Fig. 15:**
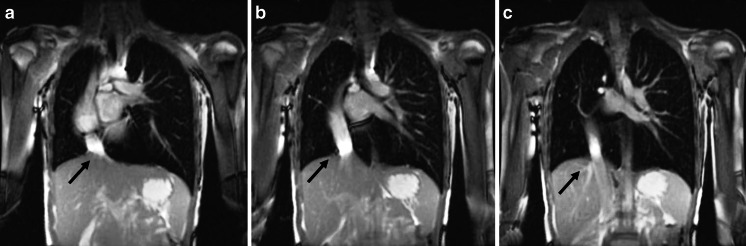
Scimitar syndrome in a 14-year-old boy. **a**-**c** Breath-hold coronal 2-D steady-state free precession demonstrates the anomalous partial pulmonary venous return (scimitar syndrome). Note the aberrant pulmonary vein connected to the inferior vena cava (*arrows*)

Non-contrast MR angiography can also be obtained with 2-D and 3-D steady-state free precession gradient echo sequences, which also supply bright blood vessel images. These sequences have been used for non-contrast evaluation of the thoracic vasculature and pulmonary embolism [40] (Fig. 15). For pulmonary embolism detection, steady-state free precession sequences showed a sensitivity of 90% for central pulmonary embolism and 80% for peripheral subsegmental pulmonary embolism [41].

### Dark blood angiography

**Fig. 16:**
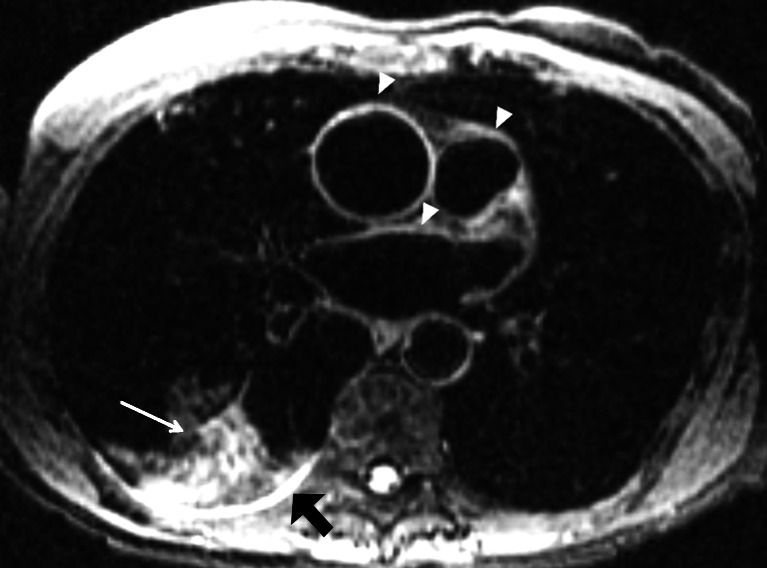
Fat-suppressed black blood fast spin echo. Axial 2-D ECG-triggered fast spin echo with black blood preparation and fat suppression in a 29-year-old man. Note the clear depiction of the mediastinal vessels (*arrowheads*), right lower lobe infiltrate (*thin arrow*) and small pleural effusion (*thick arrow*)

ECG-gated black blood single-shot fast spin echo with double inversion recovery has been used as a non-contrast sequence that provides dark vessel images and better anatomical images of the diseased lung or thoracic abnormalities (Fig. 16). Dark blood single-shot fast spin echo sequences are frequently used to study the anatomy of the heart, great vessels and mediastinum [42]. They have several advantages, including good signal-to-noise ratio, minimal motion blurring, little artifact from field inhomogeneities and superb contrast characteristics. Pulmonary emboli can be well seen using this sequence at short echo time [42].

### Contrast-enhanced MR angiography acquisitions

**Fig. 17:**
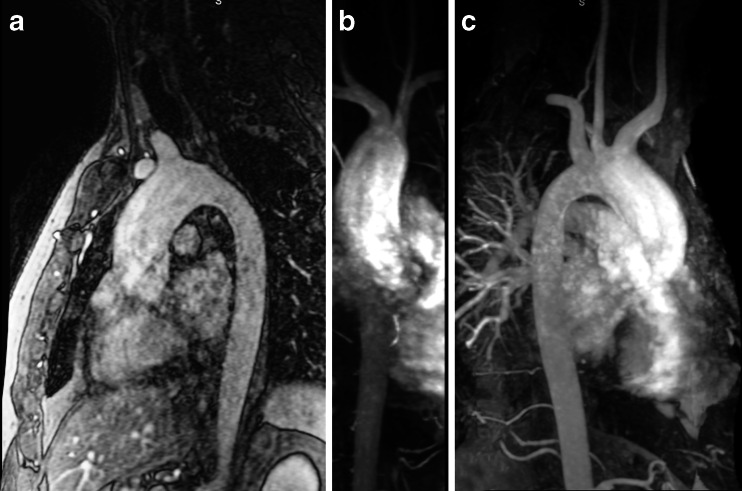
Contrast-enhanced MR angiography of the aorta in a 50-year-old man with a bicuspid aortic valve and moderate stenosis, complicated by mild insufficiency and ascending aorta dilatation. **a** T1-weighted 3-D spoiled gradient echo after a timed bolus contrast of gadolinium, **b**-**c** maximum intensity projection reconstructions

Contrast-enhanced MR angiography studies using the intravenous injection of contrast media (gadolinium chelates) are preferred over non-contrast MRI studies because they provide higher signal-to-noise ratio and shorter scan times. Contrast media provide high signal-to-noise ratio using T1-weighted scans and, hence, images can be acquired with higher temporal and spatial resolution [5]. Likewise, contrast-enhanced MR angiography renders angiographic images virtually acquired in any orientation (sagittal, coronal or axial) and is independent from inflow effects. This technique allows identification down to the sixth-order subsegmental pulmonary arteries of a normal lung [43]. Typical breath-hold MR angiography settings have very short repetition time (1.3–3 ms), short echo time (0.5–1.3 ms), and flip angles ranging between 10 and 30° depending on contrast dosage, concentration and speed of administration. Resolution and coverage are usually adjusted to suit a comfortable breath-hold [3]. In children, this technique is used to study congenital vascular anomalies, including transposition of great vessels, tetralogy of Fallot, anomalous pulmonary venous return and pulmonary sequestration (Fig. 17).

## Magnetic resonance pulmonary perfusion

Different lung diseases alter lung perfusion through the physiological mechanism of hypoxic pulmonary vasoconstriction [44]. This mechanism determines constriction of the pulmonary arteries in poorly ventilated lung areas, redirecting blood flow to normally ventilated alveoli. These reduced perfusion areas can be assessed by MRI using gadolinium, such as dynamic contrast-enhanced imaging, or without, such as arterial spin labeling [45]. Dynamic contrast-enhanced imaging has a broader use in clinical practice than arterial spin labeling, which is still used in an experimental setting. For this reason, we focus our description on dynamic contrast-enhanced imaging techniques, while discussion of arterial spin labeling is beyond the scope of this article [45].

**Fig. 18:**
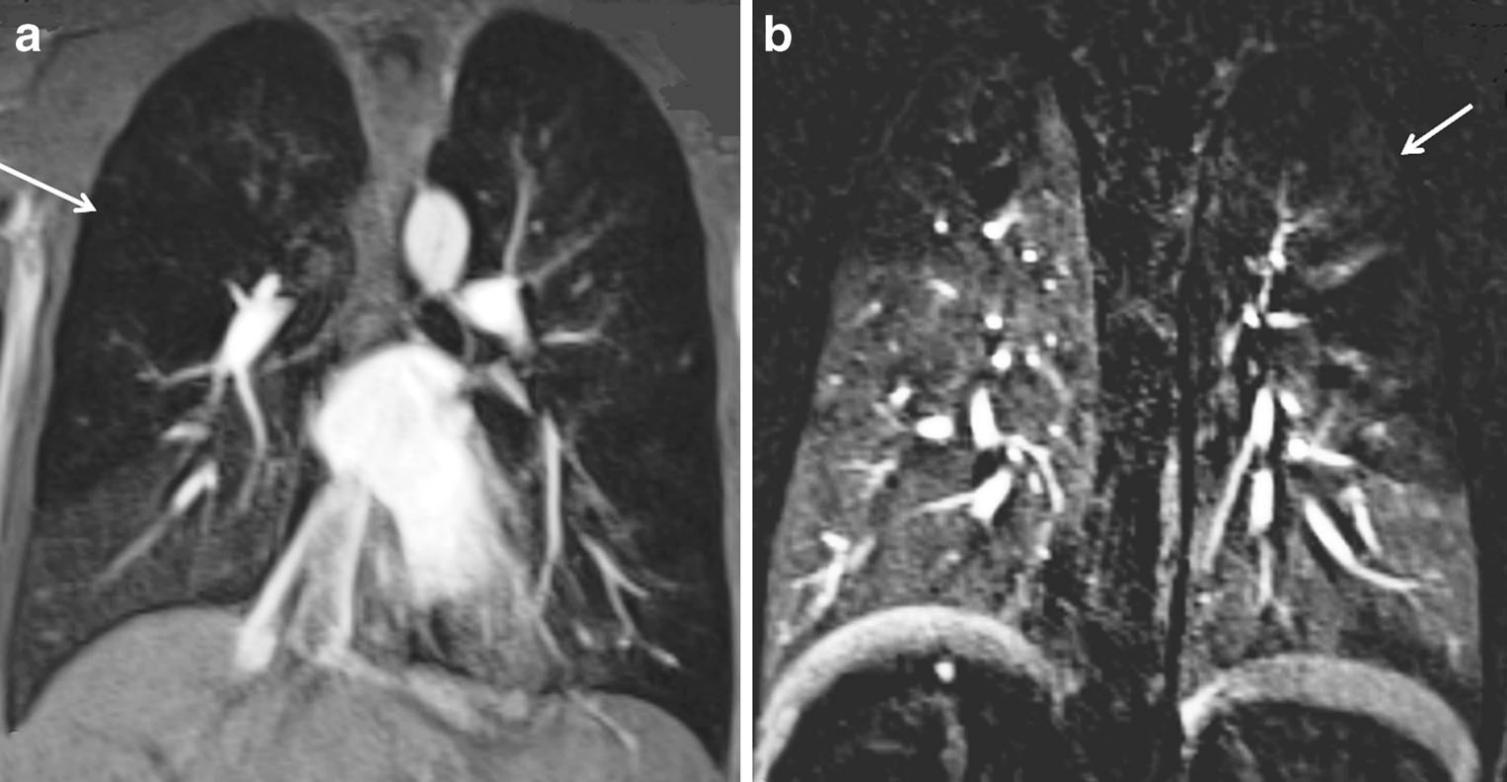
Contrast-enhanced MRI perfusion study in a 16-year-old boy with cystic fibrosis. Non-subtracted (**a**) and subtracted (**b**) coronal views. Note multiple areas of mosaic pattern likely representing areas of hypoperfusion (*arrows*)

Dynamic contrast-enhanced imaging is obtained by fast imaging of the first pass of contrast agent through the lungs after intravenous bolus injection [5]. Three-dimensional gradient echo sequences are usually preferred over 2-D technique due to the higher spatial resolution and anatomical coverage [45]. Different k-space acquisition strategies have been developed to improve temporal and spatial resolution of dynamic contrast-enhanced imaging, such as 4D-TRAK (Philips Healthcare, Best, the Netherlands), TWIST (Siemens Healthcare, Erlangen, Germany) and TRICKS (GE Healthcare, Waukesha, WI) [45]. Dynamic contrast-enhanced imaging has been largely used in adults to assess pulmonary embolism. However, it has also proved to be a very sensitive technique to assess early vascular functional impairment and therapy control in children with cystic fibrosis [9] (Fig. 18). In a study regarding childhood constrictive bronchiolitis obliterans, it was shown to be more sensitive than Tc^99m^ perfusion scintigraphy to detect perfusion defect and determine prognosis [46].

## Cine MRI

In a comparative study, good agreement was found between lung volumes as measured by pulmonary function tests and MRI [47]. In a different study, fast temporal resolution MRI techniques (cine-MRI) have been used to delineate the diaphragmatic domes and chest wall during active breathing [48]. These techniques have been proven useful for surgical and radiotherapy planning in patients with lung cancer [49]. More recently, cine MRI has been proposed to assess and monitor children after spinal surgery for scoliosis [50]. In these patients, cine MRI might be useful to understand the complex biomechanical relation between reduced vital capacity and scoliosis [50].

**Fig. 19:**
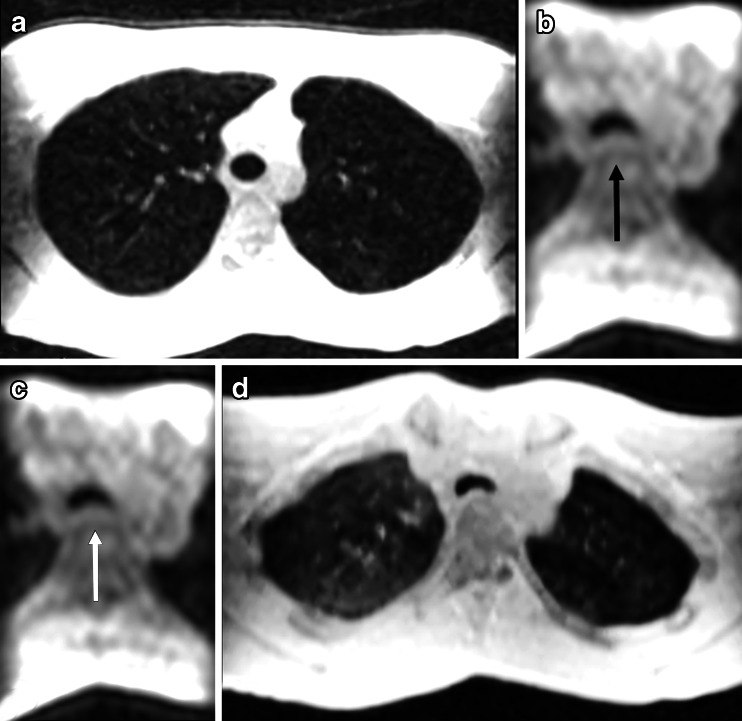
3D SPGR axial reformats at (**a**) end-inspiration, cine 3D SPGR using TRICKS acquisition axial reformat during (**b**) forced expiration, and (**c**) coughing maneuvers; (**d**) 3D SPGR axial reformats at end-expiration. Note increased tracheal collapse during breathing maneuvers (b-c, *black and white arrows*) compared to the end expiration scan (**d**)

Cine MRI has also been used to assess tracheobronchomalacia in a group of children [10]. In this study, we showed that cine MRI is a feasible technique, which might be an alternative to bronchoscopy and cine CT for tracheobronchomalacia [10] (Fig. 19). Since this study, we have routinely used MRI to assess tracheobronchomalacia in children older than 6 years.

## Hyperpolarized gases

Hyperpolarized gas MRI has demonstrated the ability to detect changes in ventilation, and lung microstructure in different pediatric lung diseases, including in asthma [51], cystic fibrosis [52], congenital diaphragmatic hernia and bronchopulmonary dysplasia [53]. However, although hyperpolarized gas MRI has exquisite sensitivity to early signs of structure-function change in pediatric lung disease, the added complexity of the technology has so far limited its use in clinical practice. Moreover, the limited availability of ^3^He has also meant that clinical uptake has been limited. However, ^129^Xe will provide a cheaper alternative for clinical lung imaging in years to come and is now ready for evaluation in pediatrics.

## Fourier decomposition

**Fig. 20:**
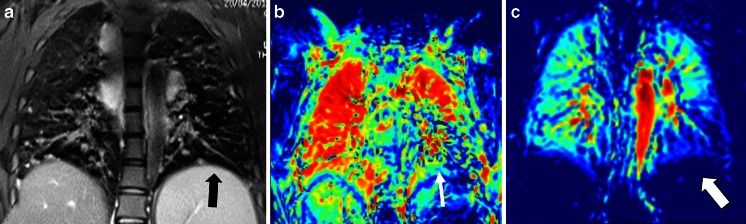
Fourier decomposition used in a 4-year-old girl with common variable immunodeficiency. **a** Radial k space-trajectory spin echo (PROPELLER), Fourier decomposition ventilation (**b**) and (**c**) perfusion generated maps. Note in (**a**) darker area in the left lower lobe (*black arrow*), which consists of an area of trapped air. In the correspondent (**b**) ventilation map that area appears not ventilated (*thin arrow*). The same area in the perfusion map (**c**) shows reduced perfusion (*thick arrow*), according to the physiological reflex of hypoxic vasoconstriction

Fourier decomposition is a new technique of non-contrast-enhanced functional lung MRI that supplies perfusion and ventilation maps not dependent on intravenous or gaseous contrast agents [54]. This new technique is based on a 2-D steady-state gradient echo sequence with high temporal resolution of 3.33 images/s acquired in coronal view without cardiac or respiratory gating. After data collection, a non-rigid image registration algorithm is applied to compensate for respiratory motion. Then, using the Fourier transformation, the signal intensity changes of the lung parenchyma related to the cardiac and respiratory cycle are decomposed to obtain the perfusion- and ventilation-weighted images [54]. For instance, Fourier decomposition has been recently applied in a group of patients with cystic fibrosis [12] and it was able to provide equivalent diagnostic information to dynamic contrast-enhanced MRI (Fig. 20).

## Conclusion

Chest MRI has reached the point where it can be used in routine clinical practice. Although MRI cannot yet be compared to CT for anatomical detail, new sequences allow acquisition of lung images with high diagnostic quality in less than 15 s, which makes MRI feasible in a clinical setting. MRI can be considered an alternative to CT for the diagnosis of lung diseases and for monitoring response to the treatment in pediatric lung disease. Moreover, in some diseases that require long-term follow-up, such as cystic fibrosis, MRI can play an important role an reducing lifelong radiation exposure related to repeated CT scans. Furthermore, MRI has the ability to offer functional information, which can only be obtained by CT at the expense of high radiation exposure. Information about lung mechanics, perfusion and ventilation can give new insight in different pediatric lung diseases. This functional information can not only improve our understanding about the pathophysiology of pediatric lung diseases, but it can also open new diagnostic and therapeutic options.
